# Congenital surfactant protein B (SP-B) deficiency: a case report

**DOI:** 10.11604/pamj.2023.44.158.35316

**Published:** 2023-04-04

**Authors:** Fatma Khalsi, Maha Chaabene, Manel Ben Romdhane, Ines Trabelsi, Samia Hamouda, Alix de Becdelièvre, Khedija Boussetta

**Affiliations:** 1 Pediatrics Department B, Children's Hospital of Tunis, Tunis, Tunisia,; 2 Department of Genetic, Henri Mondor Hospital, Genetic_Mondor, Public Assistance of the Hospitals of Paris, Delattre de Tassigny, Creteil, France

**Keywords:** SP-B deficiency, neonatal respiratory distress, lung disease, genetics, case report

## Abstract

The incapacity to synthesize certain components of pulmonary surfactant causes a heterogeneous group of rare respiratory diseases called genetic disorders of surfactant dysfunction. We report a female full-term infant with neonatal respiratory distress of early onset due to inherited SP-B deficiency. The infant failed oxygen weaning at multiple trials. Chest computed tomography was performed on the 29^th^ day of life revealing ground-glass opacities, regular interlobular septal thickening and fine interlobular reticulations. Analysis of genomic DNA showed homozygosity for an extremely rare SFTPB gene variant (c.620A>G, p.Tyr207Cys). Both parents were heterozygotes for the mutation. The diagnosis of congenital SP-B deficiency should be suspected whenever an early and acute respiratory failure in a term or near-term infant does not resolve after five days of age: diagnostic confirmation can be easily and rapidly obtained with the analysis of genomic DNA.

## Introduction

Surfactant is a combination of phospholipids and specific proteins produced by type 2 alveolar cells. It plays a major role in the maintenance of pulmonary function. The incapacity to synthesize certain components causes a heterogeneous group of rare respiratory diseases called genetic disorders of surfactant dysfunction [1,2]. They are secondary to mutations in the protein encoding genes. The clinical presentation varies with the type of disorder. Surfactant protein B deficiency was the first disorder to be discovered and it represents a rare condition [2]. We report the case of an infant that presented with persistent neonatal respiratory distress secondary to surfactant protein B deficiency.

## Patient and observation

**Patient information**: a female newborn was admitted to the pediatric department for neonatal respiratory distress. She was the product of a third-degree consanguineous marriage. The pregnancy was uneventful. The infant was delivered via emergency cesarean section at thirty-nine weeks of gestation due to meconium-stained amniotic fluid. She weighed 3500 g and had an Apgar score of 9 at 1 minute and 10 at 5 minutes.

**Clinical findings**: shortly after birth, physical examination was remarkable for tachypnea and oxygen saturation at 93%. The newborn was put under simple oxygen therapy but with no clear improvement. At 26 hours of life, she was tachypneic with a respiratory rate of 85 breaths per minute and presented with sub and intercostal retraction. The oxygen saturation dropped to 85%. The infant was transferred to the intensive care unit. Non-invasive ventilation was started but was ineffective, so the patient was intubated and put under invasive ventilation for three days. All bacterial cultures were negative. She spent two days on non-invasive ventilation and then transitioned to simple nasal oxygen cannula. The respiratory distress was initially ruled as transient tachypnea of the newborn. However, the infant failed oxygen weaning at multiple trials as she became hypoxicevery time. Infection, anemia, metabolic perturbations and cardiopathy were excluded.

**Diagnostic assessment**: chest X-rays showed worsening fine granular infiltrates. Chest computed tomography was performed on the 29^th^ day of life revealing ground-glass opacities, regular interlobular septal thickening and fine interlobular reticulations (crazy paving). These lesions were mainly localized in the posterior segments of both lungs. There was associated left lung volume loss with mediastinal shift to the ipsilateral side. Five days later, the patient benefitted from a bronchoalveolar lavage. The fluid was clear, which eliminated pulmonary alveolar proteinosis, but the analysis was inconclusive. A genetic testing for surfactant protein deficiencies was ordered for the infant and the parents. It demonstrated that the case patient was a homozygous carrier of an extremely rare SFTPB gene variant (c.620A>G, p.Tyr207Cys) that has never been described in pathology (Annex 1). The parents were heterozygous carriers. The diagnosis of surfactant protein B deficiency was confirmed.

**Diagnosis**: the diagnosis of surfactant protein B deficiency was confirmed.

**Therapeutic interventions**: at two months of age, the patient was started on monthly pulse high-dose methylprednisolone (500/m^2^ body surface area). The patient is currently 12 months old. She presents healthy weight gain and is now in the process of oxygen weaning.

**Follow-up and outcome of interventions**: chest computed tomography performed 6 months after the initiation of treatment showed significant parenchymal lesion regression. The abnormalities only persist in the inferior lobes of the caudal part of the lingula. An expansion of the left lung was noted with partial correction of the mediastinal shift.

**Patient perspective**: the pulse high-dose methylprednisolone was spaced every 3 months and was clinically well-tolerated.

**Informed consent**: the patient´s parents gave informed consent.

## Discussion

Disorders of surfactant metabolism are a diverse group of rare and complex respiratory diseases. Our case illustrates an early diagnosis of surfactant protein B deficiency in a newborn with respiratory failure. Surfactant protein B deficiency is transmitted in a recessive autosomal pattern. It is caused by a mutation of the encoding gene located on chromosome 2. Over 50 mutations have been identified: missense, nonsense, splice site and frame shift mutations [3,4]. The most common one is the 121ins2 mutation: a GAA substitution for C at genomic position g.1549 in codon 121 [5]. Our case is unique in the sense that the mutation identified is the first one described in pathology. The mutation is located at the very beginning of the functional domain found in the mature B protein. The variant is extremely rare. Only one heterozygous person has been reported in the genome ADN database. Infants with surfactant protein B deficiency present shortly after birth with respiratory distress syndrome. The clinical manifestations are similar to those of hyaline membrane disease but they usually occur in term newborns [5,6].

Chest X-rays show diffuse hazy pulmonary opacification and air bronchograms [7]. High resolution thoracic computed tomography offers more detailed images: ground-glass opacities and thickening of the intra and inter-lobular septae as a consequence of interstitial infiltrates [7]. Over the course of time, parenchymal cysts may be found indicating the progress of the disease [3]. Another integral part of the diagnostic work-up is bronchoalveolar lavage. It is performed via fibroscopy. The fluid is clear. Cytology usually shows hypercellularity with most often an increase in neutrophils. Western blot analysis identifies the abnormalities in surfactant protein expression and thus guides genetic analysis. For instance, low protein B levels indicate protein B deficiency. Low protein C levels can be found in ABCA3, protein C, protein B and TTf-1 deficiencies [3,7]. The abnormalities found on thoracic imaging, cytology and histopathology can guide the diagnosis but are not specific. Only genetic testing through DNA analysis can identify the exact mutation causing the surfactant disorder. It is guided by the clinical presentation and cytology analysis of bronchoalveolar lavage fluid [8]. Thus, results should be interpreted according to the phenotype and the genetic testing of the parents [4].

The prognosis of protein B deficiency and surfactant disorders in general is, in the vast majority of cases, rapidly unfavorable. Most infants often die within the first weeks or months of life despite the use of mechanical ventilation, exogenous surfactant therapy or extracorporeal circulation [5]. However, early diagnosis and management are crucial to limit the impact of the disease on lung growth as interstitial diseases in children occur in a developing lung. The infant in our case showed major clinical and radiological improvement thanks to early intervention. No scientifically-proven effective treatment is yet available. Currently, therapy is based on the use of anti-inflammatory drugs, mainly corticosteroids. Bolus corticosteroids are used as the first-line treatment. Oral corticosteroids are usually added initially between courses and then progressively stopped. Hydroxychloroquine and azathioprine have also been used successfully [3-6,9]. Gene replacement therapy seems to be the future therapy but it is still not fully developed [4]. Other aspects of treatment are also essential: nutritional therapy, oxygenotherapy and psychological support especially for the family.

## Conclusion

Genetic surfactant disorders are a rare group of respiratory diseases with high mortality and morbidity. They should be suspected in every newborn presenting with persistent neonatal respiratory distress or child presenting with interstitial lung disease and failure to thrive. Diagnosis is guided by imaging and bronchoalveolar lavage fluid analysis but can only be confirmed by genetic testing. Although treatment options are limited, early intervention is critical to improve diagnosis and delay the onset of pulmonary fibrosis.

## Annex

**Annex 1**: molecular findings (PDF-347ko)
